# Evaluating Large Language Models for Automated Reporting and Data Systems Categorization: Cross-Sectional Study

**DOI:** 10.2196/55799

**Published:** 2024-07-17

**Authors:** Qingxia Wu, Qingxia Wu, Huali Li, Yan Wang, Yan Bai, Yaping Wu, Xuan Yu, Xiaodong Li, Pei Dong, Jon Xue, Dinggang Shen, Meiyun Wang

**Affiliations:** 1 Department of Medical Imaging Henan Provincial People’s Hospital & People’s Hospital of Zhengzhou University Zhengzhou China; 2 Research Intelligence Department Beijing United Imaging Research Institute of Intelligent Imaging Beijing China; 3 Research and Collaboration United Imaging Intelligence (Beijing) Co, Ltd Beijing China; 4 Department of Radiology Luoyang Central Hospital Luoyang China; 5 Research and Collaboration Shanghai United Imaging Intelligence Co, Ltd Shanghai China; 6 School of Biomedical Engineering Shanghai Tech University Shanghai China; 7 Biomedical Research Institute Henan Academy of Sciences Zhengzhou China

**Keywords:** Radiology Reporting and Data Systems, LI-RADS, Lung-RADS, O-RADS, large language model, ChatGPT, chatbot, chatbots, categorization, recommendation, recommendations, accuracy

## Abstract

**Background:**

Large language models show promise for improving radiology workflows, but their performance on structured radiological tasks such as Reporting and Data Systems (RADS) categorization remains unexplored.

**Objective:**

This study aims to evaluate 3 large language model chatbots—Claude-2, GPT-3.5, and GPT-4—on assigning RADS categories to radiology reports and assess the impact of different prompting strategies.

**Methods:**

This cross-sectional study compared 3 chatbots using 30 radiology reports (10 per RADS criteria), using a 3-level prompting strategy: zero-shot, few-shot, and guideline PDF-informed prompts. The cases were grounded in Liver Imaging Reporting & Data System (LI-RADS) version 2018, Lung CT (computed tomography) Screening Reporting & Data System (Lung-RADS) version 2022, and Ovarian-Adnexal Reporting & Data System (O-RADS) magnetic resonance imaging, meticulously prepared by board-certified radiologists. Each report underwent 6 assessments. Two blinded reviewers assessed the chatbots’ response at patient-level RADS categorization and overall ratings. The agreement across repetitions was assessed using Fleiss κ.

**Results:**

Claude-2 achieved the highest accuracy in overall ratings with few-shot prompts and guideline PDFs (prompt-2), attaining 57% (17/30) average accuracy over 6 runs and 50% (15/30) accuracy with k-pass voting. Without prompt engineering, all chatbots performed poorly. The introduction of a structured exemplar prompt (prompt-1) increased the accuracy of overall ratings for all chatbots. Providing prompt-2 further improved Claude-2’s performance, an enhancement not replicated by GPT-4. The interrun agreement was substantial for Claude-2 (k=0.66 for overall rating and k=0.69 for RADS categorization), fair for GPT-4 (k=0.39 for both), and fair for GPT-3.5 (k=0.21 for overall rating and k=0.39 for RADS categorization). All chatbots showed significantly higher accuracy with LI-RADS version 2018 than with Lung-RADS version 2022 and O-RADS (*P*<.05); with prompt-2, Claude-2 achieved the highest overall rating accuracy of 75% (45/60) in LI-RADS version 2018.

**Conclusions:**

When equipped with structured prompts and guideline PDFs, Claude-2 demonstrated potential in assigning RADS categories to radiology cases according to established criteria such as LI-RADS version 2018. However, the current generation of chatbots lags in accurately categorizing cases based on more recent RADS criteria.

## Introduction

Since ChatGPT’s public release in November 2022, large language models (LLMs) have attracted great interest in medical imaging applications [1]. Research indicated that ChatGPT showed promising applications in various aspects of the medical imaging process. Even without radiology-specific pretraining, LLMs can pass board examinations [2], provide radiology decision support [3], assist in differential diagnosis [3-6], and generate impressions from findings or structured reports [7-9]. These applications not only accelerate the imaging diagnosis process and alleviate the workload of doctors but also improve the accuracy of diagnosis [10]. However, limitations exist, with 1 study showing ChatGPT-3 producing erroneous answers for a third of daily clinical questions and about 63% of provided references were not found [11]. ChatGPT’s dangerous tendency to produce inaccurate responses is less frequent in GPT-4 but still limits usability in medical education and practice at present [12]. Tailoring LLMs to radiology may enhance reliability, as an appropriateness criteria context aware chatbot outperformed generic chatbots and radiologists [12].

The American College of Radiology Reporting and Data Systems (RADS) standardizes communication of imaging findings. As of August 2023, there have been 9 disease-specific systems endorsed by the American College of Radiology, referring to products from the lexicons to report templates [13]. RADS reduces terminology variability, facilitates communication between radiologists and referring physicians, allows consistent evaluations, and conveys clinical significance to improve care. However, complexity and unfamiliarity limit adoption. Consequently, endeavors should be pursued to broaden the implementation of RADS. Therefore, we conducted this study to evaluate LLM’s capabilities on a focused RADS assignment task for radiology reports.

A prompt serves as a directive or instruction given to LLMs to generate a particular response. The technique of “prompt tuning” has emerged as a valuable approach to refine the performance of LLMs, particularly for specific domains or tasks [14]. By providing structured queries or exemplary responses, the output of chatbots can be tailored for accurate and relevant answers. Such prompt-tuning strategies leverage LLMs’ knowledge while guiding appropriate delivery for particular challenges [14]. Given the complexity and specificity of the RADS categorization, our investigation emphasizes different prompt impacts to assess chatbot capabilities and potential performance enhancement through refined prompting tuning.

In this study, our primary objective was to meticulously evaluate the performance of 3 LLMs (GPT-3.5, GPT-4, and Claude-2) for RADS categorization using different prompt-tuning strategies. We aimed to test their accuracy and consistency in RADS categorization and shed light on the potential benefits and limitations of relying on chatbot-derived information for the categorization of specific RADS.

## Methods

### Ethical Considerations

As the study was based on radiological data that were artificially generated by radiologists and did not involve the participation of human subjects, the study was determined to be exempt from ethical review, in accordance with the regulations established by the institutional review board of Henan Provincial People’s Hospital.

### Study Design

**Figure 1:**
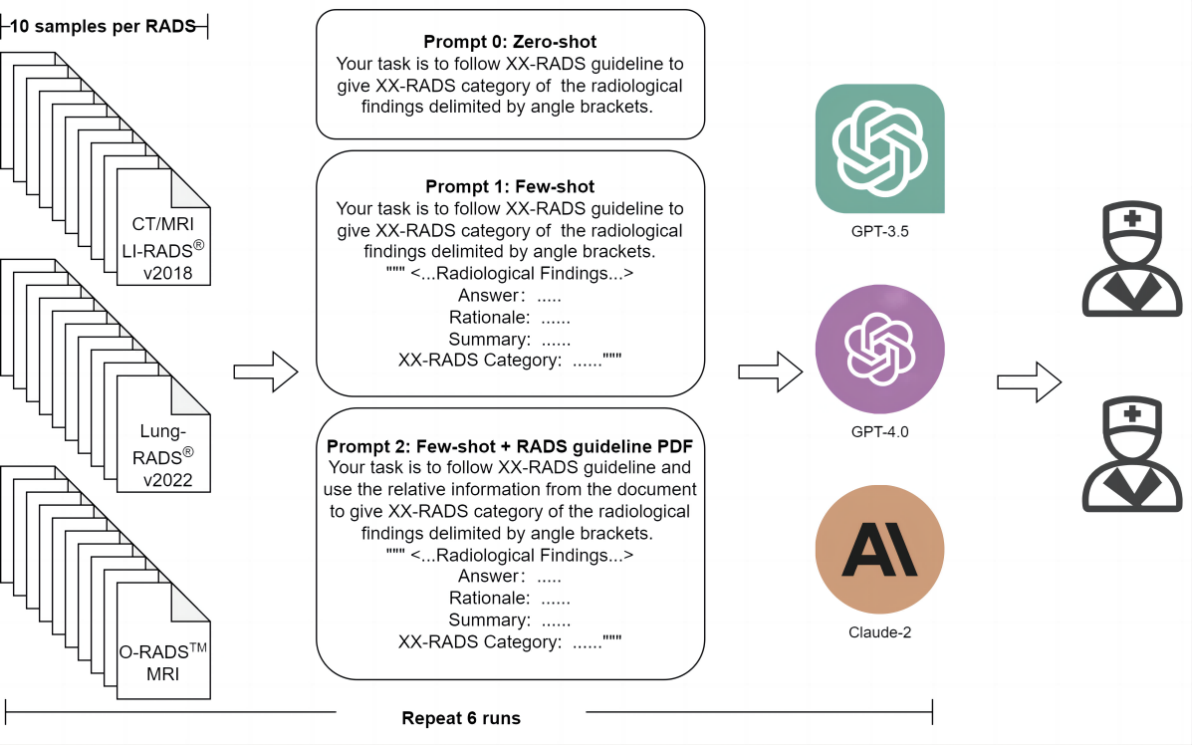
Flowchart of the study design. CT: computed tomography; LI-RADS: Liver Imaging Reporting & Data System; Lung-RADS: Lung CT Screening Reporting & Data System; MRI: magnetic resonance imaging; O-RADS: Ovarian-Adnexal Reporting & Data System; RADS: Reporting and Data Systems.

The workflow of the study is shown in Figure 1. We conducted a cross-sectional analysis in September 2023 to evaluate the competency of 3 chatbots—GPT-3.5, GPT-4 (OpenAI, August 30, 2023 version) [15], and Claude-2 (Anthropic) [16]—in the task of assigning 3 RADS categorizations to radiology reports. Given the chatbot’s knowledge cessation was as of September 2021, we opted for Liver Imaging Reporting & Data System (LI-RADS) version 2018 [17], Lung CT (computed tomography) Screening Reporting & Data System (Lung-RADS) version 2022 [18], and Ovarian-Adnexal Reporting & Data System (O-RADS) magnetic resonance imaging (MRI) (developed in 2022) [19] as the yardsticks to compare the responses engendered by GPT-3.5, GPT-4, and Claude-2. A total of thirty radiology reports for either CT or MRI examinations were composed for this analysis, with 10 cases representing each of the 3 RADS reporting systems. The radiology reports used for testing were generated by radiologists with more than 10 years’ experience to correct the wording styles from real-life cases based on respective RADS systems. For each RADS (ie, LI-, Lung-, and O-RADS), we attempted to reflect the complexity and diversity so that the reports cover typical cases in clinical practice. Therefore, reports with 2-3 simple cases and 7-8 challenging cases were generated for 1 RADS. These include scenarios such as prior examination comparison, the presence of multiple nodules, extensive categorization under different RADS systems, and updates from the most recent LI-RADS and Lung-RADS guidelines. The characteristics of radiology reports for each RADS and the distribution of the number of the reports across the 3 RADS are shown in Multimedia Appendix 1. The objective was to evaluate the performance of chatbots on a highly structured radiology workflow task involving cancer risk categorization based on structured report inputs. The study design focused on a defined use case to illuminate the strengths and limitations of existing natural language-processing technology in this radiology subdomain.

### Prompts

We collected and analyzed responses from GPT-3.5, GPT-4, and Claude-2 for each case. To mitigate bias, the radiological findings were presented individually via separate interactions, with corresponding responses saved for analysis. Three prompt templates were designed to elicit each RADS categorization along with explanatory rationale: Prompt-0 was a zero-shot prompt, merely introducing the RADS assignment task, such as “Your task is to follow Lung-RADS version 2022 guideline to give Lung-RADS category of the radiological findings delimited by angle brackets.”

Prompt-1 was a few-shot prompt, furnishing an exemplar of RADS categorization including the reasoning, summarized impression, and final category. The following is an example:

Your task is to follow Lung-RADS version 2022 guideline to give Lung-RADS category of the radiological findings delimited by angle brackets. “”“ < …Radiological Findings… > Answer：Rationale: {…} Overall: {…} Summary: {…} Lung-RADS Category: X ”“”

Prompt-2 distinctly instructed chatbots to consult the PDF of corresponding RADS guidelines, compensating for these chatbots’ lack of radiology-specific pretraining. For Claude-2, the PDF could be directly ingested, while GPT-4 required the use of an “Ask for PDF” plug-in to extract pertinent information [20,21].

Each case was evaluated 6 times with each chatbot across the 3 prompt levels. The representative radiological reports and prompts are shown in Multimedia Appendix 2. The links to all the prompts and guideline PDFs are shown in Multimedia Appendix 3.

### Evaluation of Chatbots

Two study authors (QW and HL) independently evaluated the following for each chatbot response in a blinded manner, with any discrepancies resolved by a third senior radiologist (YW). The following were assessed for each response:

1. Patient-level RADS categorization: judged as correct, incorrect, or unsure. “Correct” denotes that the chatbot accurately identified the patient-level RADS category, irrespective of the rationale provided. “Unsure” denotes that the chatbot’s response failed to provide a decisive RADS category. For example, a response articulating that “a definitive Lung-RADS category cannot be assigned” would be categorized as “unsure.”
2. Overall rating: assessed as either correct or incorrect. A response is judged incorrect if any errors (Es) are identified, including the following:
  E1: a factual extraction error that denotes the chatbots’ inability to paraphrase the radiological findings accurately, consequently misinterpreting the information.
  E2: hallucination, encompassing the fabrication of nonexistent RADS categories (E2a) and RADS criteria (E2b).
  E3: a reasoning error, which includes the incapacity to logically interpret the imaging description (E3a) and the RADS category accurately (E3b). The subtype errors for reasoning imaging description include the inability to reason lesion signal (E3ai), lesion size (E3aii), and enhancement (E3aiii) accurately.
  E4: an explanatory error, encompassing inaccurate elucidation of RADS category meaning (E4a) and erroneous explanation of the recommended management and follow-up corresponding to the RADS category (E4b).

If a chatbot’s feedback manifested any of the aforementioned infractions, it was labeled as incorrect, with the specific type of error documented. To assess the consistency of the evaluations, a k-pass voting method was also applied. Specifically, a case was deemed accurately categorized if it met the criteria in a minimum of 4 out of the 6 runs.

### Statistical Analyses

The accuracy of the patient-level RADS categorization and overall rating for each chatbot was compared using the chi-square test. The agreement across the 6 repeated runs was assessed using Fleiss κ. Agreement strength was interpreted as follows: <0 signified poor, 0-0.20 indicated slight, 0.21-0.40 represented fair, 0.41-0.60 was interpreted as moderate, 0.61-0.80 denoted substantial, and 0.81-1 was characterized as almost perfect. Statistical significance was defined as 2-sided *P*<.05. All analyses were performed using R statistical software (version 4.1.2; The R Foundation).

## Results

### Performance of Chatbots

**Figure 2:**
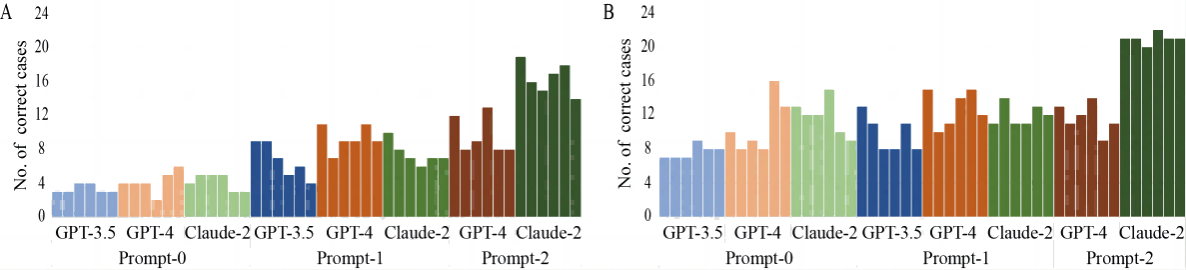
Bar graphs show the comparison of chatbot performance across 6 runs regarding (A) overall rating and (B) patient-level Reporting and Data Systems categorization.

The performance of chatbots is shown in Figure 2 and Tables 1 and 2, with the links to case-level details provided in Multimedia Appendix 4. For the overall rating (Table 1, average row and Figure 2A), Claude-2 with prompt-2 demonstrated significantly higher average accuracy across the 30 cases than Claude-2 with prompt-0 (odds ratio [OR] 8.16; *P*<.001). GPT-4 with prompt-2 also showed improved average accuracy compared with GPT-4 with prompt-0, but the difference was not statistically significant (OR 3.19; *P*=.13). When using the k-pass voting method (Table 1, k-pass voting row), Claude-2 with prompt-2 had significantly higher accuracy than Claude-2 with prompt-0 (OR 8.65; *P*=.002). Similarly, GPT-4 with prompt-2 was significantly more accurate than GPT-4 with prompt-0 (OR 11.98; *P*=.01). For the exact assignment of the patient-level RADS categorization (Table 2, average row and Figure 2B), Claude-2 with Prompt-2 showed significantly more average accuracy than Claude-2 with prompt-0 (*P*=.04).

**Table 1:** Correct overall ratings of different chatbots and prompts.

Chatbots and prompts			Prompt-0, n (%; 95% CI)	Prompt-1, n (%; 95% CI)		Prompt-2, n (%; 95% CI)
**GPT-3.5**						
	Run 1	3 (10; 3-28)		9 (30; 15-50)	N/A^a^	
	Run 2	3 (10; 3-28)		9 (30; 15-50)	N/A	
	Run 3	4 (13; 4-32)		7 (23; 11-43)	N/A	
	Run 4	4 (13; 4-32)		5 (17; 6-35)	N/A	
	Run 5	3 (10; 3-28)		6 (20; 8-39)	N/A	
	Run 6	3 (10; 3-28)		4 (13; 4-32)	N/A	
	Average^b^	3 (10; 3-28)		7 (23; 11-43)	N/A	
	K-pass voting^c^	1 (3; 0-19)		2 (7; 1-24)	N/A	
**GPT-4**						
	Run 1	4 (13; 4-32)		11 (37; 21-56)	12 (40; 23-59)	
	Run 2	4 (13; 4-32)		7 (23; 11-43)	8 (27; 13-46)	
	Run 3	4 (13; 4-32)		9 (30; 15-50)	9 (30; 15-50)	
	Run 4	2 (7; 1-24)		9 (30; 15-50)	13 (43; 26-62)	
	Run 5	5 (17; 6-35)		11 (37; 21-56)	8 (27; 13-46)	
	Run 6	6 (20; 8-39)		9 (30; 15-50)	8 (27; 13-46)	
	Average^b^	4 (13; 4-32)		9 (30; 15-50)	10 (33; 18-53)	
	K-pass voting^c^	1 (3; 0-19)		6 (20; 8-39)	9 (30; 15-50)^d^	
**Claude-2**						
	Run 1	4 (13; 4-32)		10 (33; 18-53)	19 (63; 44-79)	
	Run 2	5 (17; 6-35)		8 (27; 13-46)	16 (53; 35-71)	
	Run 3	5 (17; 6-35)		7 (23; 11-43)	15 (50; 33-67)	
	Run 4	5 (17; 6-35)		6 (20; 8-39)	17 (57; 38-74)	
	Run 5	3 (10; 3-28)		7 (23; 11-43)	18 (60; 41-77)	
	Run 6	3 (10; 3-28)		7 (23; 11-43)	14 (47; 29-65)	
	Average^b^	4 (13; 4-32)		8 (27; 13-46)	17 (57; 38-74)^d^	
	K-pass voting^c^	3 (10; 3-28)		7 (23; 11-43)	15 (50; 33-67)^d^	

^a^N/A: not applicable.

^b^Accuracy by the average method.

^c^Accuracy by k-pass voting (≥4/6 runs correct).

^d^Significant between prompt-0 and prompt-2.

**Table 2:** The number of correct, incorrect, and unsure responses for patient-level Reporting and Data Systems categorization across different chatbots and prompts.

Chatbots and prompts			Correct/incorrect/unsure patient-level Reporting and Data Systems categories, n/n/n														
			Run 1		Run 2		Run 3		Run 4		Run 5		Run 6		Average^a^		K-pass voting^b^
**GPT-3.5**																	
	Prompt-0	7/23/0		7/23/0		7/23/0		9/21/0		8/21/1		8/20/2		8/22/0		7/23/0	
	Prompt-1	13/15/2		11/19/0		8/21/1		8/21/1		11/19/0		8/22/0		10/20/0		7/23/0	
**GPT-4**																	
	Prompt-0	10/20/0		8/19/3		9/20/1		8/22/0		16/14/0		13/15/2		11/18/1		8/22/0	
	Prompt-1	15/14/1		10/18/2		11/18/1		14/15/1		15/14/1		12/18/0		13/16/1		11/19/0	
	Prompt-2	13/16/1		11/18/1		12/18/0		14/16/0		9/21/0		11/16/3		12/18/0		11/19/0	
**Claude-2**																	
	Prompt-0	13/17/0		12/18/0		12/18/0		15/15/0		10/20/0		9/21/0		12/18/0		13/17/0	
	Prompt-1	11/19/0		14/16/0		11/19/0		11/19/0		13/17/0		12/18/0		12/18/1		11/19/0	
	Prompt-2	21/9/0		21/9/0		20/10/0		22/8/0		21/9/0		2021/8/1		21/9/0^c^		21/9/0	

^a^Accuracy by the average method.

^b^Accuracy by k-pass voting (≥4/6 runs correct).

^c^Significant between prompt-0 and prompt-2.

### Consistency of Chatbots

As shown in Table 3, among the 30 cases evaluated in 6 runs, Claude-2 with prompt-2 showed substantial agreement (*k*=0.65 for overall rating; *k*=0.74 for RADS categorization). GPT-4, when interfaced with prompt-2, demonstrated moderate agreement (*k*=0.46 for overall rating; *k*=0.41 for RADS categorization). When evaluated with prompt-1, GPT-4 presented moderate agreement (*k*=0.38 for overall rating; *k*=0.42 for RADS categorization). In contrast, Claude-2 showed substantial agreement (*k*=0.63 for overall rating; *k*=0.61 for RADS categorization), while GPT-3.5 exhibited a range from slight to fair agreement. With prompt-0, Claude-2 showed moderate agreement (*k*=0.49) for overall rating and substantial agreement for RADS categorization (*k*=0.65). GPT4 manifested slight agreement (*k*=0.19) for the overall rating and fair agreement for RADS categorization. Meanwhile, GPT-3.5 showed fair agreement (*k*=0.28) for the overall rating and moderate agreement (*k*=0.57) for RADS categorization.

**Table 3:** The consistency of different chatbots and prompts among 6 runs.

			Prompt-0, Fleiss κ (95% CI)	Prompt-1, Fleiss κ (95% CI)		Prompt-2, Fleiss κ (95% CI)		All, Fleiss κ (95% CI)
**Patient-level RADS^a^** **categorization**								
	GPT-3.5	0.57 (0.48-0.65)		0.24 (0.15-0.32)	N/A^b^		0.39 (0.33-0.46)	
	GPT-4	0.33 (0.25-0.42)		0.42 (0.34-0.5)	0.41 (0.33-0.5)		0.39 (0.34-0.44)	
	Claude-2	0.65 (0.56-0.74)		0.61 (0.52-0.7)	0.74 (0.65-0.83)		0.69 (0.64-0.74)	
**Overall rating**								
	GPT-3.5	0.28 (0.19-0.37)		0.14 (0.05-0.23)	N/A		0.21 (0.14-0.27)	
	GPT-4	0.19 (0.1-0.28)		0.38 (0.29-0.47)	0.46 (0.37-0.55)		0.39 (0.34-0.45)	
	Claude-2	0.49 (0.4-0.58)		0.63 (0.53-0.72)	0.65 (0.56-0.75)		0.66 (0.61-0.72)	

^a^RADS: Reporting and Data Systems.

^b^N/A: not applicable.

### Subgroup Analysis

**Figure 3:**
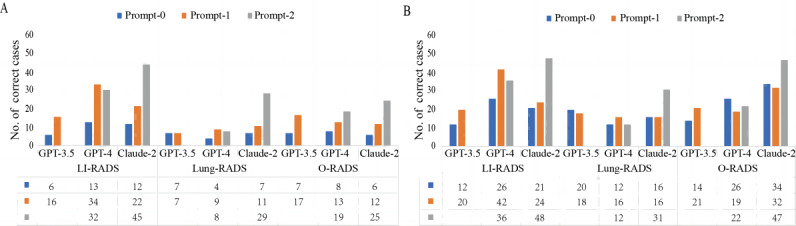
The performance of chatbots and prompts within different Reporting and Data Systems criteria. (A) Overall rating and (B) patient-level RADS categorization. LI-RADS: Liver Imaging Reporting and Data System; Lung-RADS: Lung CT (computed tomography) Screening Reporting and Data System; O-RADS: Ovarian-Adnexal Reporting and Data System.

Since the knowledge base for ChatGPT was frozen as of September 2021, accounting for the knowledge limitations of LLMs developed before the latest RADS guideline updates, we compared the responses of different RADS criteria. The total accurate responses across 6 runs were computed for all prompts. Both GPT-4 and Claude-2 demonstrated superior performance in the context of LI-RADS CT/MRI version 2018 as opposed to Lung-RADS version 2022 and O-RADS MRI (all *P*<.05; Table 4). Figure 3 delineates the performance of various chatbots across different prompts and RADS categories. For the overall rating (Figure 3A), Claude-2 exhibited a progressive trend of enhancement of overall rating accuracy from prompt-0 to prompt-1 to prompt-2, with 20.0% (12/60), 36.7% (22/60), and 75.0% (45/60) for LIRADS; 11.7% (7/60), 18.3% (11/60), and 48.3% (29/60) for Lung-RADS; and 10.0% (6/60), 20.0% (12/60), and 41.7% (25/60) for O-RADS, respectively. Notably, with prompt-2, Claude-2 achieved the highest overall rating accuracy of 75% in older systems such as LI-RADS version 2018. Conversely, GPT-4 improved with prompt-1/2 over prompt-0, but prompt-2 did not exceed prompt-1. For the RADS categorization (Figure 3B), prompt-1 and prompt-2 outperformed prompt-0 for LI-RADS, irrespective of chatbots. However, for Lung-RADS and O-RADS, prompt-0 sometimes superseded prompt-1.

**Table 4:** The performance of chatbots within different RADS criteria^a^.

Chatbots and RADS^b^			Year of development		RADS categorization (correct/incorrect/unsure), n/n/n		*P* value	Overall rating (correct/incorrect), n/n		*P* value
**GPT-3.5**										
	LI-RADS^c^ CT^d^/MRI^e^	2018		32/86/2		Reference		22/98	Reference	
	Lung-RADS^f^	2022		38/78/4		.83		14/106	.15	
	O-RADS^g^ MRI	2022		35/84/1		.46		24/96	.87	
**GPT-4**										
	LI-RADS CT/MRI	2018		104/74/2		Reference		78/102	Reference	
	Lung-RADS	2022		40/128/12		<.001		21/159	<.001	
	O-RADS MRI	2022		67/110/3		<.001		40/140	<.001	
**Claude-2**										
	LI-RADS CT/MRI	2018		93/86/1		Reference		79/101	Reference	
	Lung-RADS	2022		63/117/0		.001		47/133	<.001	
	O-RADS MRI	2022		113/67/0		.04		43/137	<.001	

^a^Data are aggregate numbers across 6 runs.

^b^RADS: Reporting and Data Systems.

^c^LI-RADS: Liver Imaging Reporting and Data System.

^d^CT: computed tomography.

^e^MRI: magnetic resonance imaging.

^f^Lung-RADS: Lung CT Screening Reporting and Data System.

^g^O-RADS: Ovarian-Adnexal Reporting and Data System.

### Analysis of Error Types

**Figure 4:**
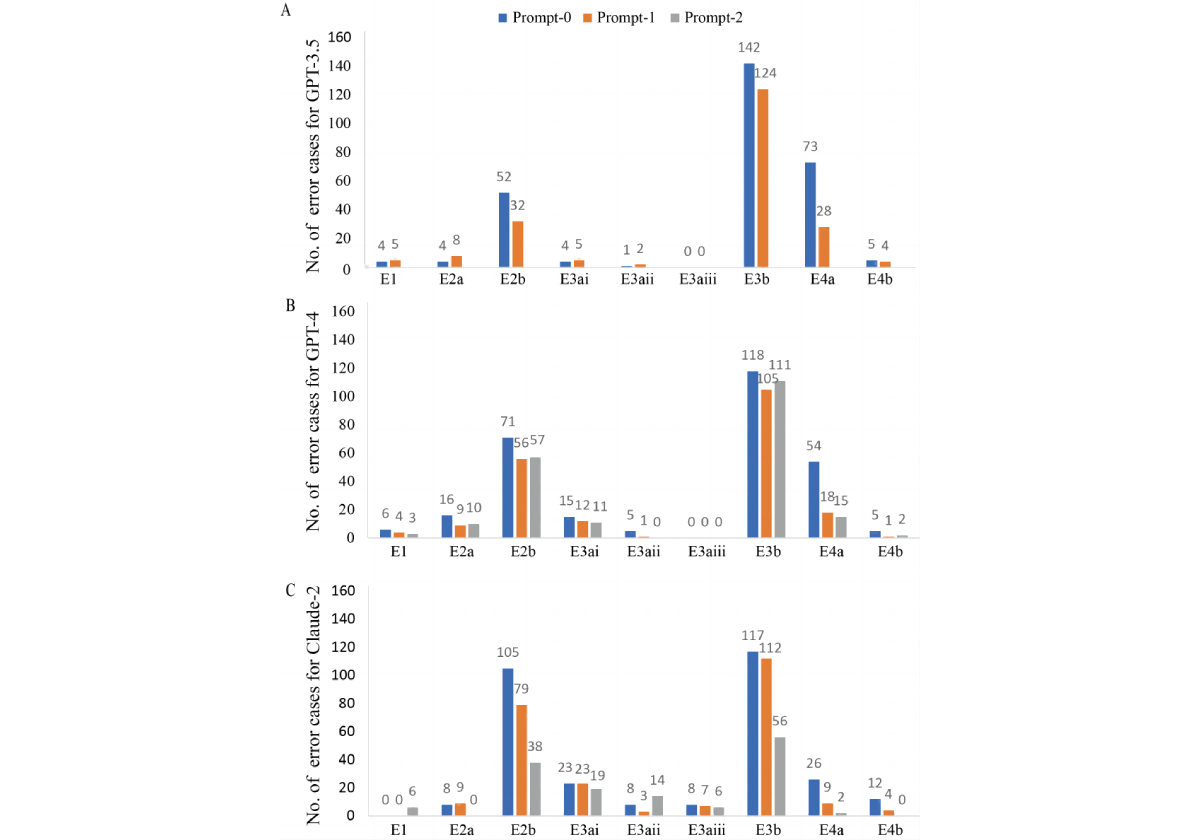
The number of error types for different chatbots. E1: Factual extraction error denotes the chatbots’ inability to paraphrase the radiological findings accurately, consequently misinterpreting the information. E2: Hallucination, encompassing the fabrication of nonexistent Reporting and Data Systems (RADS) categories (E2a) and RADS criteria (E2b). E3: Reasoning error, which includes the incapacity to logically interpret the imaging description (E3a) and the RADS category accurately (E3b). The subtype errors for reasoning imaging description include the inability to reason lesion signal (E3ai), lesion size (E3aii), and enhancement (E3aiii) accurately. E4: Explanatory error, encompassing inaccurate elucidation of RADS category meaning (E4a) and erroneous explanation of the recommended management and follow-up corresponding to the RADS category (E4b).

A total of 1440 cases were analyzed for error types, with details provided in Multimedia Appendix 4. The bar plot illustrating the distribution of errors across the 3 chatbots is shown in Figure 4. A typical example of factual extraction error (E1) occurred in response to the seventh Lung-RADS question. The statement “The 3mm solid nodule in the lateral basal segmental bronchus is subsegmental” is inaccurate, as the lateral basal segmental bronchus represents one of the 18 defined lung segments and not a subsegment [22].

Hallucination of inappropriate RADS categories (E2a) occurred more frequently with prompt-0 across all 3 chatbots. However, this error rate decreased to zero for Claude-2 when using prompt-2, a trend not seen with GPT-3.5 or GPT-4. A recurrent E2a error in LI-RADS was the obsolete category LR-5V from the 2014 version, now superseded by LR-TIV in subsequent editions [23,24]. Furthermore, hallucination of invalid RADS criteria (E2b) was more prevalent than that of E2a. For instance, the LI-RADS second question response stating “T2 marked hyperintensity is a feature commonly associated with hepatocellular carcinoma (HCC)” is inaccurate, as T2-marked hyperintensity is characteristic of hemangioma and not hepatocellular carcinoma. Despite initial higher E2b rates, Claude-2 demonstrated a substantial reduction with prompt-2 (105 to 38 instances), exceeding the decrement seen with GPT-4 (71 to 57 instances).

Regarding reasoning error, incorrect RADS category reasoning (E3b) was the most frequent error but decreased for all chatbots with prompt-1 and prompt-2 versus prompt-0. Claude-2 reduced errors by almost half with prompt-2, while the GPT-4 decrease was less pronounced. Lesion signal interpretation errors (E3ai) included misinterpreting hypointensity on diffusion-weighted imaging as “restricted diffusion,” rather than facilitated diffusion. Lesion size reasoning errors (E3aii) occurred in 34 of 1440 cases, predominantly by Claude-2 (25/34, 73.5%), especially in systems such as Lung-RADS and LI-RADS where size is critical for categorization. Examples were attributing a 12-mm pulmonary nodule to the ≥6-mm but <8-mm range, or assigning a hepatic lesion measuring 2.3 cm × 1.5 cm to the 10- to 19-mm category. Reasoning enhancement errors (E3aiii) were exclusive to Claude-2 in O-RADS, where enhancement significantly impacts categorization. Misclassifying images at 40 seconds postcontrast as early or delayed enhancement exemplifies this error.

Explanatory errors (E4) including incorrect RADS category definitions (E4a) and inappropriate management recommendations (E4b) also substantially declined with prompt-1 and prompt-2. For instance, in the first Lung-RADS question response, the statement “The 4X designation indicates infectious/inflammatory etiology is suspected.” is incorrect. Lung-RADS 4X means category 3 or 4 nodules with additional features or imaging findings that increase suspicion of lung cancer [18].

## Discussion

### Principal Findings

In this study, we evaluated the performance of 3 chatbots—GPT-3.5, GPT-4, and Claude-2—in categorizing radiological findings according to RADS criteria. Using 3 levels of prompts providing increasing structure, examples, and domain knowledge, the chatbots’ accuracies and consistencies were quantified across 30 cases. The best performance was achieved by Claude-2 when provided with few-shot prompting and the RADS criteria PDFs. Interestingly, the chatbots tended to categorize better for the relatively older LI-RADS version 2018 criteria in contrast to the more recent Lung-RADS version 2022 and O-RADS guidelines published after the chatbots’ training cutoff.

The incorporation of RADS, which standardizes reporting in radiology, has been a significant advancement, although the multiplicity and complexity of these systems impose a steep learning curve for radiologists [13]. Even for subspecialized radiologists at tertiary hospitals, mastering the numerous RADS guidelines poses challenges, requiring familiarity with the lexicons, regular application in daily practice, and ongoing learning to remain current with new versions. While previous studies have shown that LLMs could assist radiologists in various tasks [2-5,7,11], their performance at RADS categorization from imaging findings is untested. We therefore evaluated LLMs for focused RADS categorization of testing cases.

Without prompt engineering (prompt-0), all chatbots performed poorly. However, accuracy improved for all chatbots when provided an exemplar prompt demonstrating the desired response structure (prompt-1). This underscores the use of prompt tuning for aligning LLMs to specific domains such as radiology. Further enriching prompt-1 with the RADS guideline PDFs as a relevant knowledge source (prompt-2) considerably enhanced Claude-2’s accuracy, a feat not mirrored by GPT-4. This discrepancy could stem from ChatGPT’s reliance on an external plug-in to access documents, while Claude-2’s architecture accommodates the direct assimilation of expansive texts, benefiting from its larger-context window and superior long document–processing capabilities.

Notably, we discerned performance disparities across RADS criteria. When queried on older established guidelines such as LI-RADS version 2018 [17], the chatbots demonstrated greater accuracy than more recent schemes such as Lung-RADS version 2022 and O-RADS [18,19,25]. Specifically, GPT-4 and Claude-2 had significantly higher total correct ratings for LI-RADS than for Lung-RADS and O-RADS (all *P*<.05). This could be attributed to their extensive exposure to the voluminous data related to the matured LI-RADS during their pretraining phase. With prompt-2, Claude-2 achieved 75% (45/60) accuracy for overall rating LI-RADS categorization. The poorer performance on newer RADS criteria highlights the need for strategies to continually align LLMs with the most up-to-date knowledge.

A deep dive into the error-type analysis revealed informative trends. Incorrect RADS category reasoning (E3b) constituted the most frequent error across chatbots, decreasing with prompt tuning. Targeted prompting also reduced critical errors such as hallucinations of RADS criteria (E2b) and categories (E2a) likely by constraining output to valid responses. During pretraining, GPT-liked LLMs predict the next word in the unlabeled data set, risking learning fallacious relationships between RADS features. For instance, Lung-RADS version 2022 lacks categories 5 and 6 [18], though some other RADS such as Breast Imaging Reporting and Data System include them [26]. Using prompt-0, chatbots erroneously hallucinated Lung-RADS categories 5 and 6. Explanatory errors (E4) including inaccurate definition of the assigned RADS category (E4a) and inappropriate management recommendations (E4b) also substantially declined with prompt tuning. For instance, when queried on the novel O-RADS criteria with prompt-0, chatbots hallucinated follow-up recommendations from other RADS criteria and responded “O-RADS category 3 refers to an indeterminate adnexal mass and warrants short-interval follow-up.” Targeted prompting appears to mitigate these critical errors such as hallucination and incorrect reasoning. Careful prompt engineering is essential to properly shape LLM knowledge for radiology tasks.

### Limitations

There are also several limitations in this study. First, only the LI-RADS CT/MRI and O-RADS MRI were included, excluding LI-RADS ultrasound (US) and O-RADS US guidelines, which are often practiced in an independent US department [27,28]. Second, the chatbot’s performance was heavily dependent on prompt quality. We test only 3 types of prompts and further prompt strategies studies are warranted to investigate the impact of more exhaustive engineering on chatbots’ accuracy. Third, GPT-4-turbo was released on November 6, 2023, representing the latest GPT-4 model with improvements in instruction following, reproducible outputs, and more [29]. Furthermore, its training data extend to April 2023 compared with September 2021 for the base GPT-4 model tested here. We are uncertain about this newest GPT-4-turbo model’s performance on the RADS categorization task. Evaluating GPT-4-turbo represents an important direction for future work. Fourth, our study focused on 3 of 9 RADS [13], with a limited 10 cases for each RADS category. Although our choice ensured a blend of old and new guidelines and tried to cover all the RADS scores as much as possible, extending evaluations to all the RADS guidelines and incorporating more radiology reports from real clinical scenarios could offer deeper insights into potential limitations. Nonetheless, this initial study highlights critical considerations of prompt design and knowledge calibration required for safely applying LLMs in radiology. Fifth, evaluating the performance of the LLM in comparison with radiologists of varying expertise levels proves valuable for discerning its strengths and weaknesses in real-world applications. This comparative analysis will be undertaken in our forthcoming studies.

### Conclusions

When equipped with structured prompts and guideline PDFs, Claude-2 demonstrates potential in assigning RADS categories to radiology cases according to established criteria such as LI-RADS version 2018. However, the current generation of chatbots lags in accurately categorizing cases based on more recent RADS criteria. Our study highlights the potential of LLMs in streamlining radiological categorizations while also pinpointing the enhancements necessary for their dependable application in clinical practice for RADS categorization tasks.

## Appendix

Multimedia Appendix 1 The characteristics of radiology reports for each of the Reporting and Data Systems (RADS) and the distribution of the number of the reports across the 3 RADS.

Multimedia Appendix 2 Representative radiology reports and prompts.

Multimedia Appendix 3 Links to prompts and guideline PDFs.

Multimedia Appendix 4 Links to prompt engineering results.

## Abbreviations

CT: computed tomography
E: error
LI-RADS: Liver Imaging Reporting & Data System
LLM: large language model
Lung-RADS: Lung CT Screening Reporting & Data System
MRI: magnetic resonance imaging
O-RADS: Ovarian-Adnexal Reporting & Data System
OR: odds ratio
RADS: Reporting and Data Systems
US: ultrasound
