# Supplementary material for: Evaluating Large Language Models for Automated Reporting and Data Systems Categorization: Cross-Sectional Study
Source: JMIR Med Inform. 2024 Jul 17;12:e55799. doi: 10.2196/55799 (PMC11292156; doi:10.2196/55799)

# Multimedia Appendix 1

**Table 1** The characteristics of radiology reports for each RADS

|  | LI-RADS | Lung-RADS | O-RADS |
| --- | --- | --- | --- |
| Comparison to prior exams |  |  |  |
| Yes | 4 | 5 | - |
| No | 6 | 5 | - |
| Nodularity |  |  |  |
| Single | 9 | 7 | 10 |
| Multiple | 1 | 3 | 0 |
| Scenarios |  |  |  |
| Diagnose category | 8 | 10 | 10 |
| Treatment response category | 2 | - | - |
| Distribution of RADS category |  |  |  |
| Incomplete | 0 | 1 | - |
| Definitely benign or malignant | 3 | 2 | 2 |
| Probably benign or malignant | 5 | 7 | 8 |
| Treatment response | 2 | - | - |

Note. “-” denotes not applicable.

**Figure 1. The distribution of the number of the reports across LI-RADS, Lung-RADS, and O-RADS.** A) LI-RADS category. B) patient-level Lung-RADS category. C) nodule-level Lung-RADS category. D) O-RADS category.


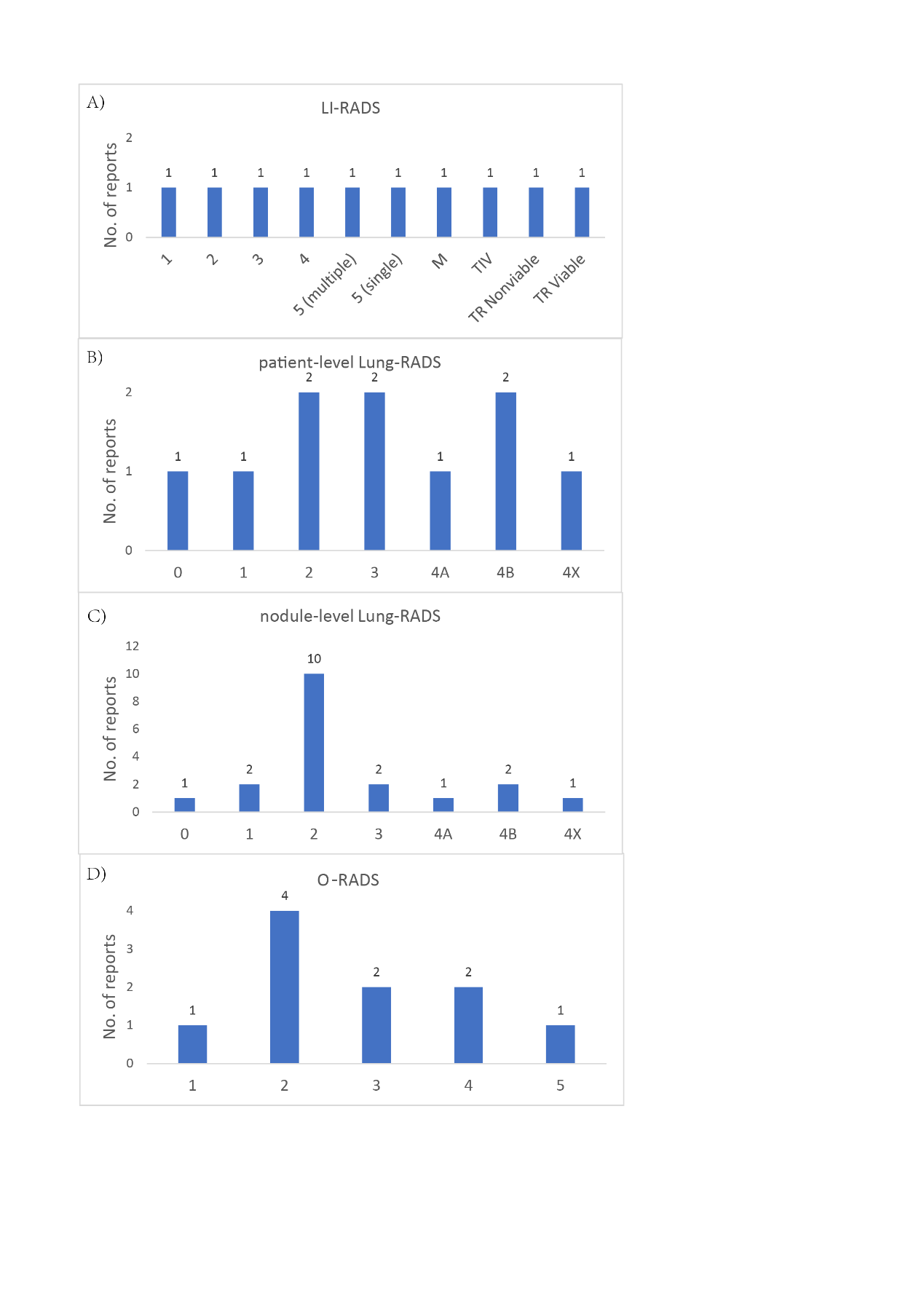

Supplement: Multimedia Appendix 1 [file medinform_v12i1e55799_app1.docx]
