# Supplementary material for: Evaluating Large Language Models for Automated Reporting and Data Systems Categorization: Cross-Sectional Study
Source: JMIR Med Inform. 2024 Jul 17;12:e55799. doi: 10.2196/55799 (PMC11292156; doi:10.2196/55799)
Supplement: Multimedia Appendix 2 [file medinform_v12i1e55799_app2.docx]

# Multimedia Appendix 2

## Representative radiology reports and prompts.

### Prompt-0 for GPT-3.5, GPT-4 and Claude-2

Your task is to follow Lung-RADS® v2022 guideline to give Lung-RADS category of the radiological findings delimited by angle brackets.

<CT scan of the chest WITHOUT intravenous contrast, using low-dose lung cancer screening protocol.

**COMPARISON:** None

**FINDINGS:**

**Lines/tubes:** None.

**Lungs and Airways:** Multiple part-solid nodules and consolidations are seen in the posterior segments of left upper lung and dorsal segment of the left lower lobe.

**Pleura:** The pleural spaces are clear.

**Base of neck, mediastinum and heart:** The thyroid gland is normal. No mediastinal, hilar or axillary lymphadenopathy is seen. The heart and pericardium are within normal limits.

**Soft tissues:** Normal.

**Abdomen:** This study was performed without contrast and with lower than standard dose. These factors reduce the sensitivity for detection of small lesions in the upper abdomen. Given these technical limitations, no focal lesion is seen within the visualized liver, spleen, pancreas, kidneys and adrenal glands.

**Bones:** The visualized bony thorax is within normal limits.>

### Prompt-1 for GPT-3.5, GPT-4 and Claude-2

Your task is to follow Lung-RADS® v2022 guideline to give Lung-RADS category of the radiological findings delimited by angle brackets.

**"""**

<CT scan of the chest WITHOUT intravenous contrast, using low-dose lung cancer screening protocol.

**COMPARISON:** LDCT 6 months ago

**FINDINGS:**

**Lines/tubes:** None.

**Lungs and Airways:**

There are multiple solid nodules in bilateral lungs, the largest solid nodule is located in the right upper lobe and the size is 7mm, these nodules are similar to those on LDCT 6 months ago. The solid nodule in the left upper lobe is newly emerging, and the size is 5mm. There is a part-solid nodule in the right upper lobe, the total diameter is 7mm and the diameter of the solid component is 5mm, this part-solid nodule is similar to that on LDCT 6 months ago.

**Pleura:** The pleural spaces are clear.

**Base of neck, mediastinum and heart:** The thyroid gland is normal. No mediastinal, hilar or axillary lymphadenopathy is seen. The heart and pericardium are within normal limits.

**Soft tissues:** Normal.

**Abdomen:** This study was performed without contrast and with lower than standard dose. These factors reduce the sensitivity for detection of small lesions in the upper abdomen. Given these technical limitations, no focal lesion is seen within the visualized liver, spleen, pancreas, kidneys and adrenal glands.

**Bones:** The visualized bony thorax is within normal limits.>

**Answer：**

**Rationale:**

- Multiple solid nodules, the largest solid nodule is 7mm, which is Lung-RADS 3, while it is stable compared to prior LDCT 6 months ago, it can be downgraded from Lung-RADS 3 to Lung-RADS 2.
- The new emerging solid nodule is 5mm in size, which is larger than 4mm, smaller than 6mm, so it is Lung-RADS 3.
- A part-solid nodule, the diameter is 7mm, larger than 6mm and the solid component is 5mm, less than 6mm, so it is Lung-RADS 3, and it is stable compared to prior LDCT 6 months ago, it can be downgraded from Lung-RADS 3 to Lung-RADS 2.

**Overall:**

Considering all the above nodules, the nodule with the biggest Lung-RADS should be assigned as this patient’s Lung-RADS. So the Lung-RADS of this patient is Lung-RADS 3.

**Summary:**

A new 5mm solid nodule in the left upper lobe, Lung-RADS 3;

part-solid nodule in the right upper lobe, stable, Lung-RADS 2;

Multiple solid nodules identified in both lungs with the largest one of 7mm, stable, Lung-RADS 2;

**Lung-RADS Category: 3**

"""

<CT scan of the chest WITHOUT intravenous contrast, using low-dose lung cancer screening protocol.

**COMPARISON:** None

**FINDINGS:**

**Lines/tubes:** None.

**Lungs and Airways:** Multiple part-solid nodules and consolidations are seen in the posterior segments of left upper lung and dorsal segment of the left lower lobe.

**Pleura:** The pleural spaces are clear.

**Base of neck, mediastinum and heart:** The thyroid gland is normal. No mediastinal, hilar or axillary lymphadenopathy is seen. The heart and pericardium are within normal limits.

**Soft tissues:** Normal.

**Abdomen:** This study was performed without contrast and with lower than standard dose. These factors reduce the sensitivity for detection of small lesions in the upper abdomen. Given these technical limitations, no focal lesion is seen within the visualized liver, spleen, pancreas, kidneys and adrenal glands.

**Bones:** The visualized bony thorax is within normal limits.>

### Prompt-2 for GPT-4

Document ID: 909b17e7-be12-47e1-adf5-b1d1dde293e9

Your task is to follow Lung-RADS® v2022 guideline and use the relative information from the document to give Lung-RADS category of the radiological findings delimited by angle brackets.

**"""**

<CT scan of the chest WITHOUT intravenous contrast, using low-dose lung cancer screening protocol.

**COMPARISON:** LDCT 6 months ago

**FINDINGS:**

**Lines/tubes:** None.

**Lungs and Airways:**

There are multiple solid nodules in bilateral lungs, the largest solid nodule is located in the right upper lobe and the size is 7mm, these nodules are similar to those on LDCT 6 months ago. The solid nodule in the left upper lobe is newly emerging, and the size is 5mm. There is a part-solid nodule in the right upper lobe, the total diameter is 7mm and the diameter of the solid component is 5mm, this part-solid nodule is similar to that on LDCT 6 months ago.

**Pleura:** The pleural spaces are clear.

**Base of neck, mediastinum and heart:** The thyroid gland is normal. No mediastinal, hilar or axillary lymphadenopathy is seen. The heart and pericardium are within normal limits.

**Soft tissues:** Normal.

**Abdomen:** This study was performed without contrast and with lower than standard dose. These factors reduce the sensitivity for detection of small lesions in the upper abdomen. Given these technical limitations, no focal lesion is seen within the visualized liver, spleen, pancreas, kidneys and adrenal glands.

**Bones:** The visualized bony thorax is within normal limits.>

**Answer：**

**Rationale:**

- Multiple solid nodules, the largest solid nodule is 7mm, which is Lung-RADS 3, while it is stable compared to prior LDCT 6 months ago, it can be downgraded from Lung-RADS 3 to Lung-RADS 2.
- The new emerging solid nodule is 5mm in size, which is larger than 4mm, smaller than 6mm, so it is Lung-RADS 3.
- A part-solid nodule, the diameter is 7mm, larger than 6mm and the solid component is 5mm, less than 6mm, so it is Lung-RADS 3, and it is stable compared to prior LDCT 6 months ago, it can be downgraded from Lung-RADS 3 to Lung-RADS 2.

**Overall:**

Considering all the above nodules, the nodule with the biggest Lung-RADS should be assigned as this patient’s Lung-RADS. So the Lung-RADS of this patient is Lung-RADS 3.

**Summary:**

A new 5mm solid nodule in the left upper lobe, Lung-RADS 3;

part-solid nodule in the right upper lobe, stable, Lung-RADS 2;

Multiple solid nodules identified in both lungs with the largest one of 7mm, stable, Lung-RADS 2;

**Lung-RADS Category: 3**

"""

<CT scan of the chest WITHOUT intravenous contrast, using low-dose lung cancer screening protocol.

**COMPARISON:** None

**FINDINGS:**

**Lines/tubes:** None.

**Lungs and Airways:** Multiple part-solid nodules and consolidations are seen in the posterior segments of left upper lung and dorsal segment of the left lower lobe.

**Pleura:** The pleural spaces are clear.

**Base of neck, mediastinum and heart:** The thyroid gland is normal. No mediastinal, hilar or axillary lymphadenopathy is seen. The heart and pericardium are within normal limits.

**Soft tissues:** Normal.

**Abdomen:** This study was performed without contrast and with lower than standard dose. These factors reduce the sensitivity for detection of small lesions in the upper abdomen. Given these technical limitations, no focal lesion is seen within the visualized liver, spleen, pancreas, kidneys and adrenal glands.

**Bones:** The visualized bony thorax is within normal limits.>

### Prompt-2 for Claude-2

Your task is to follow Lung-RADS® v2022 guideline and use the relative information from the document to give Lung-RADS category of the radiological findings delimited by angle brackets.

**"""**

<CT scan of the chest WITHOUT intravenous contrast, using low-dose lung cancer screening protocol.

**COMPARISON:** LDCT 6 months ago

**FINDINGS:**

**Lines/tubes:** None.

**Lungs and Airways:**

There are multiple solid nodules in bilateral lungs, the largest solid nodule is located in the right upper lobe and the size is 7mm, these nodules are similar to those on LDCT 6 months ago. The solid nodule in the left upper lobe is newly emerging, and the size is 5mm. There is a part-solid nodule in the right upper lobe, the total diameter is 7mm and the diameter of the solid component is 5mm, this part-solid nodule is similar to that on LDCT 6 months ago.

**Pleura:** The pleural spaces are clear.

**Base of neck, mediastinum and heart:** The thyroid gland is normal. No mediastinal, hilar or axillary lymphadenopathy is seen. The heart and pericardium are within normal limits.

**Soft tissues:** Normal.

**Abdomen:** This study was performed without contrast and with lower than standard dose. These factors reduce the sensitivity for detection of small lesions in the upper abdomen. Given these technical limitations, no focal lesion is seen within the visualized liver, spleen, pancreas, kidneys and adrenal glands.

**Bones:** The visualized bony thorax is within normal limits.>

**Answer：**

**Rationale:**

- Multiple solid nodules, the largest solid nodule is 7mm, which is Lung-RADS 3, while it is stable compared to prior LDCT 6 months ago, it can be downgraded from Lung-RADS 3 to Lung-RADS 2.
- The new emerging solid nodule is 5mm in size, which is larger than 4mm, smaller than 6mm, so it is Lung-RADS 3.
- A part-solid nodule, the diameter is 7mm, larger than 6mm and the solid component is 5mm, less than 6mm, so it is Lung-RADS 3, and it is stable compared to prior LDCT 6 months ago, it can be downgraded from Lung-RADS 3 to Lung-RADS 2.

**Overall:**

Considering all the above nodules, the nodule with the biggest Lung-RADS should be assigned as this patient’s Lung-RADS. So the Lung-RADS of this patient is Lung-RADS 3.

**Summary:**

A new 5mm solid nodule in the left upper lobe, Lung-RADS 3;

part-solid nodule in the right upper lobe, stable, Lung-RADS 2;

Multiple solid nodules identified in both lungs with the largest one of 7mm, stable, Lung-RADS 2;

**Lung-RADS Category: 3**

"""

<CT scan of the chest WITHOUT intravenous contrast, using low-dose lung cancer screening protocol.

**COMPARISON:** None

**FINDINGS:**

**Lines/tubes:** None.

**Lungs and Airways:** Multiple part-solid nodules and consolidations are seen in the posterior segments of left upper lung and dorsal segment of the left lower lobe.

**Pleura:** The pleural spaces are clear.

**Base of neck, mediastinum and heart:** The thyroid gland is normal. No mediastinal, hilar or axillary lymphadenopathy is seen. The heart and pericardium are within normal limits.

**Soft tissues:** Normal.

**Abdomen:** This study was performed without contrast and with lower than standard dose. These factors reduce the sensitivity for detection of small lesions in the upper abdomen. Given these technical limitations, no focal lesion is seen within the visualized liver, spleen, pancreas, kidneys and adrenal glands.

**Bones:** The visualized bony thorax is within normal limits.>
