# Supplementary material for: Evaluating Large Language Models for Automated Reporting and Data Systems Categorization: Cross-Sectional Study
Source: JMIR Med Inform. 2024 Jul 17;12:e55799. doi: 10.2196/55799 (PMC11292156; doi:10.2196/55799)
Supplement: Multimedia Appendix 3 [file medinform_v12i1e55799_app3.docx]

# Multimedia Appendix 3

## Links to Prompts.

<https://dalomegoxbn.feishu.cn/docx/TaFpdeHvRoFFTExQgvnchfeQnW5?from=from_copylink> OR

<https://docs.google.com/document/d/1MNWQJ6NoQ_cNJwR6BKLbXF2icw6Zh0bM/edit?usp=sharing&ouid=109358782966595828845&rtpof=true&sd=true>

## Links to guideline PDFs.

## LungRADS v2022: <https://dalomegoxbn.feishu.cn/file/Ktaub2Az0oSJtjx5NVtcVrfinAg?from=from_copylink> OR <https://drive.google.com/file/d/1w31vV705Q4ncRpERBzykLdMmQJ0nduLg/view?usp=sharing>

## LI-RADS CT/MRI v2018: <https://dalomegoxbn.feishu.cn/file/GSvsbFjv3oNXbqxogEsczrRCnTe?from=from_copylink> OR <https://drive.google.com/file/d/1bSVWqGD3zZi-5xoyQDCXZSXyWtsi3JZm/view?usp=sharing>

## O-RADS MRI: <https://dalomegoxbn.feishu.cn/file/Ck6ib47XFoyhVFxZkwDcJtH8ncc> OR <https://drive.google.com/file/d/1panirRsjbu6w_8MQPeOtTAlDjxTzFZBz/view?usp=sharing>
