# Supplementary material for: Evaluating Large Language Models for Automated Reporting and Data Systems Categorization: Cross-Sectional Study
Source: JMIR Med Inform. 2024 Jul 17;12:e55799. doi: 10.2196/55799 (PMC11292156; doi:10.2196/55799)
Supplement: Multimedia Appendix 4 [file medinform_v12i1e55799_app4.docx]

# Multimedia Appendix 4

## Links to Prompt Engineering Results.

<https://dalomegoxbn.feishu.cn/sheets/COXmsLNK6hNJN9t1xXccnqhJnRc?from=from_copylink> OR

<https://docs.google.com/spreadsheets/d/1nXqEbV7sH_G8QOblqLG_OEJ1xH6PzYHB/edit?usp=sharing&ouid=109358782966595828845&rtpof=true&sd=true>
